# IgA nephropathy to proliferative glomerulonephritis with monoclonal IgAκ deposits: a pattern switch

**DOI:** 10.1007/s40620-023-01583-2

**Published:** 2023-03-13

**Authors:** Jin-pu Li, Ya-ting Du, Chuan Guo, Xiang-rong Rao, Shen Li

**Affiliations:** Renal Division, Guang’anmen Hospital, China Academy of Chinese Medical Sciences, No. 5 Beixiange St. Xicheng District, Beijing, People’s Republic of China

**Keywords:** Proliferative glomerulonephritis with monoclonal immunoglobulin deposits, Membranoproliferative glomerulonephritis, Refractory nephrotic syndrome, IgA nephropathy, Monoclonal gammopathy of renal significance

## Abstract

We report the case of a 31-year-old male who presented with repeated episodes of nephritic-nephrotic syndrome in concomitance with infection. IgA was diagnosed and was initially responsive to treatment with immunosuppressors but further disease flare did not respond to treatment. Based on three consecutive renal biopsies over 8 years, a pattern switch from endocapillary proliferative IgA nephropathy to membranous proliferative glomerulonephritis with monoclonal IgAκ deposits was observed. Bortezomib-dexamethasone combination therapy finally led to a favorable renal response. This case provides new insights into the pathophysiological mechanisms of proliferative glomerulonephritis with monoclonal immunoglobin deposits (PGNMID), highlighting the importance of repeat renal biopsies and routine evaluation of monoclonal immunoglobin deposits in proliferative glomerulonephritis with refractory nephrotic syndrome.

## Introduction

Proliferative glomerulonephritis with monoclonal immunoglobin deposits (PGNMID) is a distinct entity in which a monoclonal immunoglobin is deposited in the glomerulus, mimicking immune complex-mediated proliferative glomerulonephritis [1], such as endocapillary proliferative glomerulonephritis (EPGN) and membranoproliferative glomerulonephritis (MPGN). Monoclonal immunoglobin deposit-related glomerulopathy may also occur with a membranous, cryoglobulinemic, amyloidosic, or crescentic pattern [2]. Membranoproliferative glomuerulonephritis is present in approximately 2/3 of reported cases and endocapillary proliferative glomerulonephritis is associated with 20–35% of PGNMID cases [3]. Monoclonal immunoglobin deposition or C3 glomerulonephritis may initially manifest as endocapillary proliferative glomerulonephritis and then progress to membranoproliferative glomerulonephritis, which is more common in immunoglobin deposition, and/or complement activation.

Proliferative glomerulonephritis with monoclonal IgA deposits is seen mostly in the setting of indolent plasma cell disorders [4], which rarely induce IgA nephropathy (IgAN) [5]. Additionally, primary IgA nephropathy is characterized by the frequently predominant λ over κ light chain deposition.

We present a rare case of IgA nephropathy with an endocapillary proliferative pattern detected on the first and second kidney biopsies, which then switched to a membranoproliferative glomerulonephritis pattern and was ultimately diagnosed as IgA2κ-PGNMID on the third kidney biopsy. The patient presented with nephritic-nephrotic syndrome and preceding infectious symptoms. He was successfully treated with bortezomib plus dexamethasone and achieved a favorable renal response.

## Case report

A 31-year-old Chinese male presented with acute kidney injury and nephrotic syndrome, with prodromal symptoms of upper respiratory-tract infection for one week. He had a past medical history of hypertension and nephritic syndrome. In 2013, he underwent kidney biopsy due to nephrotic syndrome at a different institution. The biopsy demonstrated endocapillary proliferative IgA nephropathy (M1E1S0T1C0), immunofluorescence (IF) staining showed strong deposition of IgA and C3. Further information about the first biopsy was unavailable. The patient was treated with losartan, prednisone and leflunomide, followed by tripterygium glycosides, leading to complete remission. However, he developed progressive proteinuria and was admitted to our institution in 2017. On admission, physical examination was unremarkable except for mild lower limb edema. Laboratory investigations showed serum creatine 380 μmol/L, serum albumin 2.39 g/dL, and hemoglobin 7.6 g/dL. Urinalysis showed 37.26 RBCs/HPF with 90% dysmorphic RBCs, and urinary protein excretion 11.3 g/d. Serum C3 level was low at 0.542 g/L (Table 1).

**Table 1:** Results of consecutive renal biopsies and laboratory tests

Time point	2013, biopsy 1 at a different institution	2017, biopsy 2 at our institution	2019, follow-up	2020, biopsy 3 at our institution	2021, follow-up
Biopsy diagnosis	Endocapillary proliferative-IgAN	Endocapillary proliferative-IgAN and subacute tubulointerstitial nephritis	–	IgA2κ-PGNMID	–
Renal function	SCr 140 μmol/L (reference range 44–130 μmol/L), UP 5.5 g/d	SCr 380 μmol/L, UP 11.3 g/d	SCr 100 μmol/L, UP 4.8 g/d	SCr 216 μmol/L, UP 9.1 g/d	SCr 140.7 μmol/L, UP 0.348 g/d
Serum C3	Not available	0.542 g/L (reference range 0.9–1.8 g/L)	0.499 g/L	0.544 g/L	0.97 g/L
Monoclonal work-up	Not available	n/a	SIFE positive for IgAκ Bone marrow biopsy showed a few plasma cells with normal change Flow cytometry CD38-gating analysis of bone marrow aspiration revealed 21.2% CD38-positive κ monotypic plasma cells expressed CD138, CD56, CD27, CD117, and were negative for CD19, CD20, CD5, CD23 and CD28	S/U IFE positive for IgAκ Flow cytometry on bone marrow aspiration revealed 72.4% CD38-positive κ monotypic plasma cells expressed CD138, CD56, CD27, and were negative for CD19, CD117, CD20, CD5, CD23 and CD28 SFLC κ light chain 347 mg/dL (reference range 33–194 mg/dL), λ light chain 358 mg/dL (reference range 57–264 mg/dL), κ/λ 0.97 (reference range 0.37–3.17)	SIFE, SPEP negative; sFLC κ light chain 178 mg/dL, λ light chain 210 mg/dL, κ/λ 0.85
Infection work-up	Not available	ASO, HBV, HCV, syphilis and HIV negative	HBV, HCV, syphilis and HIV negative	ASO, HBV, HCV, syphilis and HIV negative	Parvovirus B19 IgG antibody positive
Others	Not available	n/a	Cryoglobulin test negative	Anti-dsDNA antibody, anti-GBM antibody and ANCA-negative	Normal CH50; CFH antibody-negative
Treatment	RASI, PRED + LEF	RASI, PRED + CTX	RASI, PRED + CTX followed by PRED + AZA + HCQ	RASI, initial treatment with Bortezomib + Dexamethasone	RASI, after five cycles of Bortezomib + Dexamethasone

*ASO* Antistreptolysin O, *ANCA* anti-neutrophil cytoplasmic antibody, *AZA* azathioprine, *anti-GBM* anti-glomerular basement membrane, *CFH* complement factor H, *CTX* cyclophosphamide, *EPGN* endocapillary proliferative glomerulonephritis, *FLC* free light chain, *HBV* hepatitis B virus, *HCQ* hydroxychloroquine, *HCV* hepatitis C virus, *HIV* human immunodeficiency virus, *IgAN* IgA nephropathy, *LEF* leflunomide, *MPGN* membranoproliferative glomerulonephritis, *n/a* not applicable, *PGNMID* proliferative GN with monoclonal immunoglobin deposits, *PRED* prednisolone, *RASI* renin–angiotensin system inhibition, *S/U IFE* serum/urine immunofixation, *SCr* serum creatine, *UP* urinary protein excretion

**Fig. 1:**
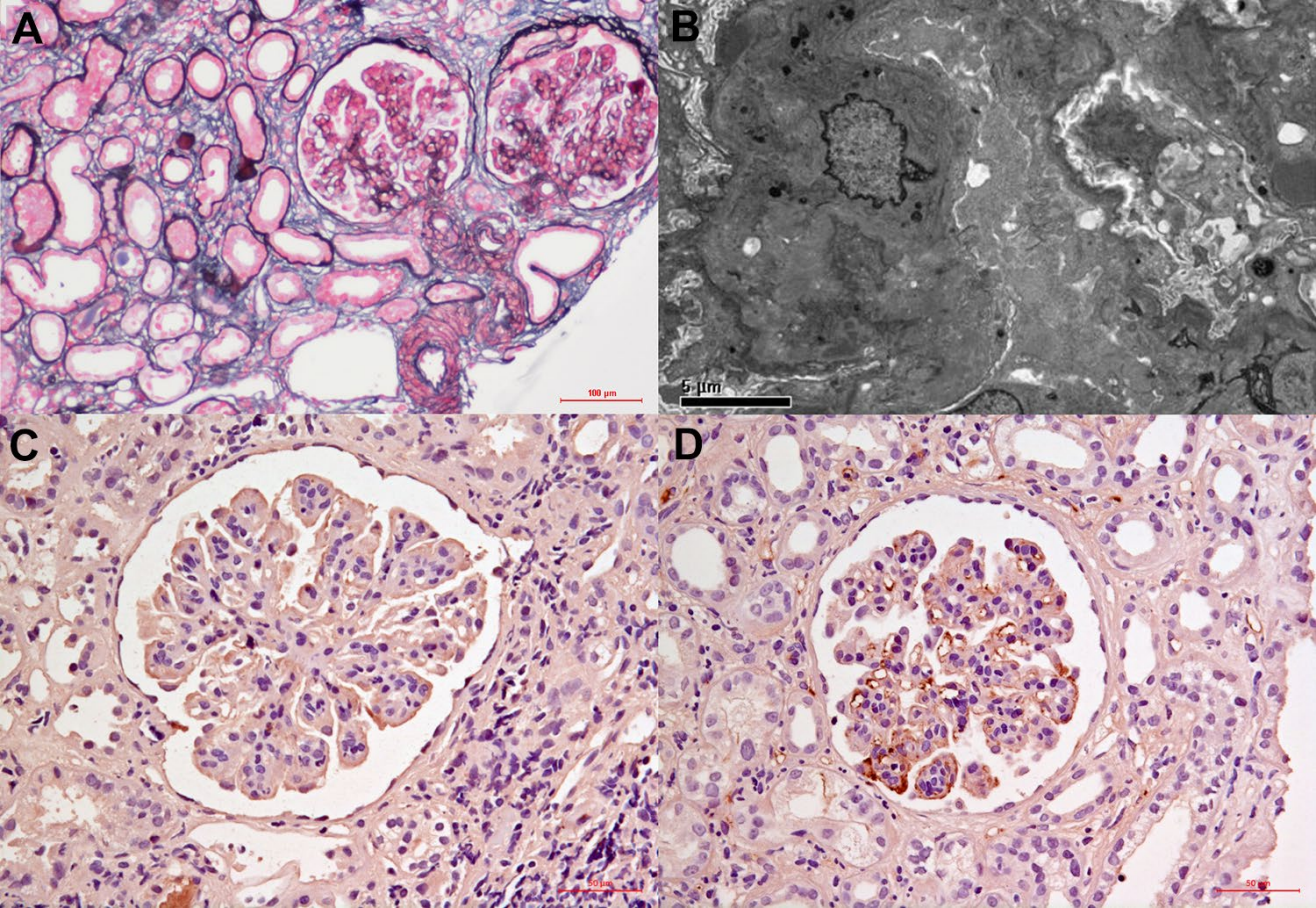
Findings on the second kidney biopsy. Light Microscopy showed **A** Endocapillary proliferative glomerulonephritis (periodic methenamine silver and Masson trichrome staining; original magnification, × 200). Electron Microscopy showed **B** electron-dense deposits in subepithelial, basement membrane and mesangial area, with segmental intramembranous dense ribbon-like deposition (× 5000). **C** Negative IHC staining for IgA1, with **D** positive staining for IgA2 (× 400)

Hence, the patient underwent a second kidney biopsy. Immunofluorescence demonstrated granular IgA (2 +) and C3 (3 +) deposition in the mesangium and along the capillary wall, with negative IgG, IgM, C1q and C4d (figure not shown). Light microscopy showed 14/24 glomeruli with endocapillary proliferative glomerulonephritis pattern (Fig. 1A). Electron microscopy revealed multiple electron-dense deposits and segmental intramembranous dense ribbon-like deposition (Fig. 1B). Endocapillary proliferative IgA nephropathy (M1E1S1T2C1) and subacute tubulointerstitial nephritis was diagnosed. The patient was given prednisolone and cyclophosphamide to treat IgA nephropathy. Serum creatinine decreased to 100–130 μmol/L and proteinuria decreased to 0.65 g/d six months after treatment start, while hemoglobin levels returned to normal. One year later, the disease flared in the wake of an oral infection but resolved with prednisolone and losartan, prednisone and leflunomide. However, the disease reactivated after withdrawal of losartan and leflunomide treatment while still on prednisolone and an angiotensin-converting enzyme inhibitor. Thereafter, further therapy (prednisolone and mycophenolate mofetil or cyclophosphamide) did not lead to remission. A monoclonal IgAκ was identified in serum through immunofixation electrophoresis. Bone marrow biopsy showed normal plasma cells. Bone marrow aspiration revealed 21.2% plasma cells with aberrant phenotype. Plasma cells were κ light chain restricted on flow cytometry. Cryoglobulins were absent. Retrospective immunohistochemical staining for light chain on paraffin-embedded tissue following proteinase digestion of the second renal biopsy was negative for both κ and λ light chain. Since there was no evidence of monoclonal immunoglobulin-related renal lesions or extrarenal involvement, our diagnosis was monoclonal gammopathy of undetermined significance, and we continued immunosuppression therapy. The patient was then treated with prednisolone plus azathioprine and hydroxychloroquine. By this point, proteinuria had decreased from 11.4 to 4.8 g/d.

In the third year of follow-up, the disease relapsed with edema and monoclonal gammopathy. Laboratory examinations showed the following: serum creatine 216 μmol/L, proteinuria 9.1 g/d, serum albumin 2.86 g/dL, hemoglobin 9.4 g/dL, C3 0.544 g/L, urine protein test (+). Hematological investigation showed an IgAκ in both serum and urine immunoelectrophoresis; serum protein electrophoresis showed decreased gamma globulin at 10.17%; bone marrow aspiration was unchanged, bone marrow flow cytometry immunofluorescence assay indicated 72.4% immunophenotypic abnormal plasma cells with κ light chain restricted expression; serum free light chain assay showed κ light chain 347 mg/dL, λ light chain 358 mg/dL, kappa to lambda ratio (κ/λ) was within normal range at 0.97.

**Fig. 2:**
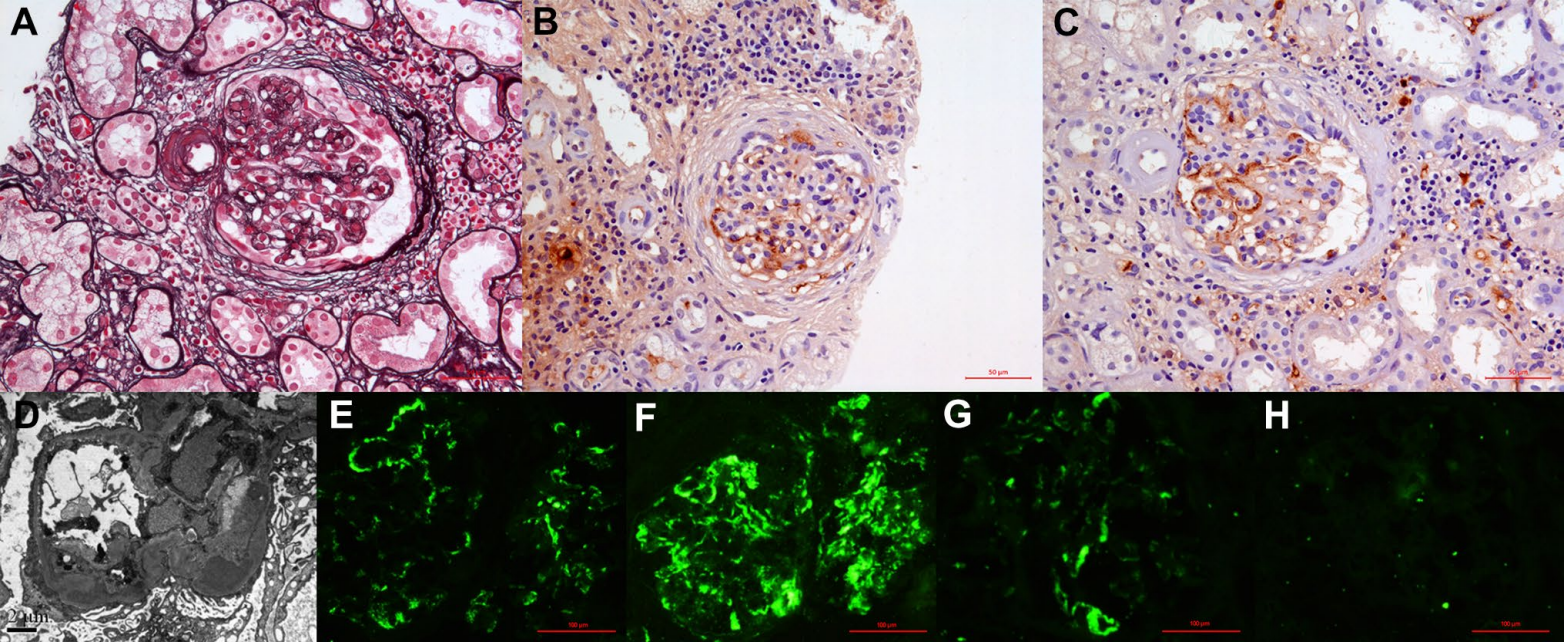
Findings on the third kidney biopsy. Light microscopy showed **A** typical membranoproliferative glomerulonephritis pattern (× 400). **B** Negative immunohistochemical staining for IgA1, with **C** positive staining for IgA2 (× 400). Electron microscopy showed **D** electron-dense deposits in the mesangial and subendothelial area, double contouring of the glomerular capillary walls and mesangial interposition with extensive foot process effacement (× 8000). Immunofluorescence  showed intense glomerular positivity for **E** granular IgA and **F** C3 deposition in mesangium, with strong mesangial staining for **G** kappa light chains and negative staining for **H** lambda light chains (× 400)

A third renal biopsy was performed. Immunofluorescence showed IgA (2 +  ~ 3 +), C3 (3 +) and κ light chain (2 +  ~ 3 +) deposits in nodular and granular patterns predominantly in mesangium (Fig. 2E, F and G), with negative λ light chain (Fig. 2H), IgM, IgG, IgG1-4, C1q, FRA, and albumin (figure not shown). Light microscopy showed diffuse mesangial matrix proliferation with segmental interposition of mesangial cells; endocapillary hypercellularity with neutrophil infiltration; segmental platinum ear-pick, double contour formation along the glomerular basement membrane. Compared with the second biopsy, there was more prominent tubular atrophy, greater interstitial fibrosis, and inflammatory cell infiltration in multiple parts of the interstitial region (Fig. 2A). Electron microscopy revealed electron-dense deposits in the subendothelial and mesangial areas, segmented double contours and moth-eaten lesions of the glomerular basement membrane, and extensive foot process effacement (Fig. 2D). At the ultrastructural level, the deposits were not fibrillary or microtubular. Congo red staining, immunohistochemical staining for amyloid A protein, C4d, CD138 were negative. Immunohistochemical staining for IgA subclasses demonstrated that IgA2 ( +) deposits were localized to the mesangium and along the capillary wall in nodular and granular patterns, with negative staining for IgA1 (Fig. 2B, C). We also performed immunohistochemical staining for IgA subclasses on the second renal biopsy and found the same result as the third biopsy (Fig. 1C, D). These pathological and clinical features suggest that a change had occurred in the phenotype of immunoglobulins during follow-up. Finally, a diagnosis of IgA2κ-PGNMID was considered. The patient was started on the first of five cycles of a scheme combining bortezomid and betamethasone. Further examinations were then performed to explore the etiology and pathogenesis of PGNMID. Complement Factor H antibody was negative and CH50 was within the normal range. C3 nephritic factor was not measured. However, parvovirus B19 IgG antibody was positive with an index of 13 (normal range < 1.1). Other chronic infections were not identified.

The treatment gains were stable at 1-year follow-up, which included resolved kidney disease (serum creatine, 140.7 μmol/L; serum albumin, 5.07 g/dL; hemoglobin, 14.7 g/dL; urinary protein, 347.5 mg/d; urine protein test (+)), and hematological remission (negative serum immunofixation electrophoresis; κ serum free light chain 178 mg/dL, λ serum free light chain 210 mg/dL, κ/λ 0.85). Serum C3 level was within normal range at 0.97 g/L.

## Discussion

On the basis of immunofluorescence findings, proliferative glomerulonephritis can be classified as (1) immune complex or immunoglobulin (Ig)-mediated proliferative glomerulonephritis with or without C3 deposition, such as IgAN/post-infection glomerulonephritis with predominant IgA deposition and monoclonal immunoglobulin-associated proliferative glomerulonephritis; (2) pauci-immune proliferative glomerulonephritis; and (3) complement-mediated proliferative glomerulonephritis without or with only minimal  deposition of immunoglobulins [6]. The pathological pattern of proliferative glomerulonephritis changes depending on the course of the disease and the time point of biopsy. Our patient had a protracted course and underwent three renal biopsies over 8 years, characterized by nephritic-nephrotic syndrome with impaired renal function. His condition worsened when infections occurred and immunosuppressive agents were withdrawn. Both of the first kidney biopsy findings were membranoproliferative glomerulonephritis-IgAN, which then switched to MPGN-PGNMID. Post-infectious glomerulonephritis triggered by bacterial infections (such as group A streptococci and staphylococcus) often present as endocapillary proliferative glomerulonephritis, which is typically characterized by polyclonal Ig with intense granular C3 deposition along the capillary wall and subepithelial hump-like electron-dense deposits. In the present case, immunofluorescence showed consistently strong staining for IgA and C3 in all three renal biopsies with negative C1q and C4d, suggesting activation of alternative pathway of complement system. However, repeated Antistreptolysin O test results were negative, and electron microscopy findings were not consistent with a diagnosis of IgA-dominant post-infectious glomerulonephritis. The presence of parvovirus B19 IgG antibody was indicative of prior exposure. The history of parvovirus B19 infection may have thus played a role in disease progression. Autoantibodies or exogenous antigenic stimulus (viral or bacterial infection), leading to the trapping or in situ formation of circulating immune complexes, may show pathological patterns consistent with post-infectious glomerulonephritis. Infection-induced immune response to exogenous antigens may be involved in PGNMID development, presumably through a mechanism related to B cell repertoire remodeling, while stimulating the differentiation of polyclonal B cells to plasma cells which may also lead to a switch in pathological type as the disease progresses. Moreover, the patient responded poorly to immunosuppression therapy for IgAN, which could partly support our hypothesis. Yu et al. [7] reported the evolution of an immune complex-related membranoproliferative glomerulonephritis switch to PGNMID in a patient with a history of HCV infection. However, the influence of long-term immunosuppression on the function and population of plasma cells regarding long-lived immunoglobin production was not investigated.

Monoclonal IgA1-λ deposition is found in light- and heavy-chain deposition disease, cryoglobulinemia, immune tentacle-like glomerulonephritis, and PGNMID [4]. IgA-PGNMID is rare (< 5%) among PGNMIDs, and tends to progress to multiple myeloma. Approximately 1/3 of cases of IgA-PGNMID are initially misdiagnosed as IgAN. Consecutive immunohistochemical staining for IgA subclasses of this case showed positive IgA2 and negative IgA1. IgA is divided into IgA1 and IgA2, which are primarily distinguished by the structure of the hinge region and the number of glycosylation sites. IgA O- and N-glycosylation profiles may influence complement activation, interaction with bacteria, and clearance of circulating IgA and immune complexes [8]. Compared with IgA1, the physicochemical properties of IgA2 are more stable against bacterial proteases [9]. IgA2 is more pro-inflammatory and exhibits a higher degree of salivary acidification and galactosylation than those of IgA1 [10]. Notably, kidney cells or receptors involved in IgA2 clearance and catabolism are rarely reported.

It is reasonable to consider infection as a breakthrough in the mechanism of disease evolution in this case. It can be hypothesized that recurrent infections during the disease course may have depleted circulating and mucosal IgA1 and progressively amplified aberrant complement alternative pathway regulation while inducing the appearance of traces of monoclonal immunoglobulins. In contrast, IgA1 immune complexes in renal tissue were digested and broken down by renal cells with immunosuppressive treatment and time, and thus the level of monoclonal IgA2 in circulation and renal tissues gradually increased, demonstrating a transient dense deposit disease pattern on electron microscopy and progressive endocapillary proleferative glomerulonephritis with increasing interstitial fibrosis and tubular atrophy on light microscopy. Concurrently, the concentration of circulating monoclonal immunoglobulin was still below the detection limit of immunofixation electrophoresis, protein electrophoresis, and other routine tests. The disease ultimately progressed to PGNMID. The pre-treatment serum free light chain assay was within the normal range, and proteinuria was within nephrotic range, whereas proteinuria was lower than the detection limit by dry chemistry urinalysis. This also supports the possibility that IgA2k, an intact monoclonal immunoglobulin, may be involved in PGNMID as a minor autoantibody to complement alternative pathway regulatory protein or C3 convertase.

The treatment of PGNMID is controversial owing to limited cases and uncertain pathogenesis. Among patients with PGNMID in whom clones were identified (approximately 30%), 50% had plasmacytoid clones and 50% had clones derived from B lymphocytes [11]. In our patient, since the results of bone marrow flow cytometry assays showed an apparent pathological plasma cell clone, therapy with bortezomid and dexamethasone was used to induce remission, which appeared to optimally balance efficacy and patient tolerability [4, 12].

In summary, to our knowledge, this is the first case of IgA2-PGNMID switching from endocapillary proliferative-IgAN characterized by recurrent infection throughout the protracted course of disease. A plasma cell-targeted regimen based on bortezomib plus dexamethasone induced sustained renal and hematologic response. We propose that the pathological type can shift as the disease itself progresses due to exogenous immune challenges such as infection and local complement activation. This case also highlights the need for repeat renal biopsies and routine evaluation of monoclonal immunoglobin deposit-related proliferative glomerulonephrits  with refractory nephrotic syndrome, for diagnosis and appropriate therapy prescription.

## Data Availability

All data generated or analysed during this study are included in this published article.
